# Content Analysis of Arabic Websites As Patient Resources for Osteoporosis

**DOI:** 10.7759/cureus.64880

**Published:** 2024-07-19

**Authors:** Dalal M Alabdulmohsen, Mesa A Almahmudi, Ahmed I Alnajjad, Adnan M Almarzouq

**Affiliations:** 1 Internal Medicine, College of Medicine, King Faisal University, Al-Ahsa, SAU; 2 Endocrinology, Dr. Sulaiman Al-Habib Hospital, Al-Khobar, SAU

**Keywords:** reliability, readability, patient education, educational websites, osteoporosis, quality analysis

## Abstract

Background: Osteoporosis is a prevalent metabolic bone disease in the Middle East. Middle Easterners rely on the Internet as a source of information about osteoporosis and its treatment. Adequate awareness can help to prevent osteoporosis and its complications. Websites covering osteoporosis in Arabic must be of good quality and readability to be beneficial for people in the Middle East.

Methods: Two Arabic terms for osteoporosis were searched on Google.com (Google Inc., Mountainview, CA), and the first 100 results for each term were examined for eligibility. Two independent raters evaluated the websites using DISCERN and the Journal of the American Medical Association (JAMA) criteria for quality and reliability. The Flesch Kincaid grade level (FKGL), Simple Measure of Gobbledygook (SMOG), and Flesch Reading Ease (FRE) scale were used to evaluate the readability of each website’s content.

Results: Twenty-five websites were included and evaluated in our study. The average DISCERN score was 28.36±12.18 out of 80 possible scores. The average JAMA score was 1.05±1.15 out of four total scores. The readability scores of all websites were, on average, 50.71±21.96 on the FRE scale, 9.25±4.89 on the FKGL, and 9.74±2.94 on the SMOG. There was a significant difference (p = 0.026 and 0.044) in the DISCERN and JAMA scores, respectively, between the websites on the first Google page and the websites seen on later pages.

Conclusion: The study found Arabic websites covering osteoporosis to be of low quality and difficult readability. Because these websites are a major source for patient education, improving their quality and readability is a must. The use of simpler language is needed, as is covering more aspects of the diseases, such as prevention.

## Introduction

Osteoporosis is the most prevalent bone disease worldwide, affecting nearly one in five elderly people globally [1,2]. A progressive decrease in bone mass density characterizes this condition, resulting in skeletal fragility and an increased susceptibility to fractures. These fractures are the main cause of the illness and disability associated with the disease [3]. There are many etiologies behind osteoporosis, the most common one being estrogen deficiency-related bone loss, which explains why postmenopausal women are the predominant group of patients. Other secondary causes include vitamin D deficiency, hyperparathyroidism, and drugs such as steroids [4].

Management of the disease typically involves lifestyle modifications, minerals and vitamin supplements, and antiresorptive medical therapy [5]. It is worth noting that osteoporosis can, in fact, be prevented. Although most cases are not detected until the late stages of the disease, patients who are recognized early can benefit from interventions that reduce and slow down bone loss, including diet, exercise, and behavioral changes [6]. Even for patients in later stages with irreversible bone changes, strategies to prevent fractures are crucial [6]. It is believed that good knowledge about osteoporosis may motivate at-risk patients to engage in preventative measures to minimize bone deterioration [7]. Therefore, being well aware of the condition and having access to reliable information can contribute to improving the health outcomes of the disease.

A study was conducted in 2023 by Saleh A. et al. to measure the knowledge of osteoporosis in different Arab countries in the Middle East and North Africa (MENA). In this study, the most common source that Arabs used for information regarding osteoporosis was the Internet [8]. The Internet has become the primary platform for health information internationally. However, a major drawback is the vast amount of unregulated content, raising concerns about its overall quality and reliability. This presents a significant challenge for users without medical expertise who struggle to evaluate the credibility of online health resources [9].

While the online content covering osteoporosis and related aspects in English was assessed by researchers, the Arabic online content has not yet been evaluated [10-13]. Notably, Arabic is the most spoken language in the Middle East region, which has a higher incidence of low bone mass than Western countries [14]. Hence, this study intends to fill this gap in the literature and offer a qualitative assessment of Internet content directed towards Arabic patients' education on the disease.

## Materials and methods

For this study, we used the search engine Google (http://www.google.com, Google Inc., Mountainview, CA) on April 8, 2024, with Google Chrome version 124.0.6367.91 to look up results for the Arabic term for osteoporosis, “هشاشة العظام (*hashashat aleizam*),” and an alternative term for the same disease, “الهشاشة (*al hashshah*).” Google was chosen because it is the most popular search engine worldwide and occupies about 92% of the market share [15]. Moreover, we deleted cookie information and search history, and we used “incognito” (private) mode for browsing to avoid bias from previous Internet use.

**Figure 1:**
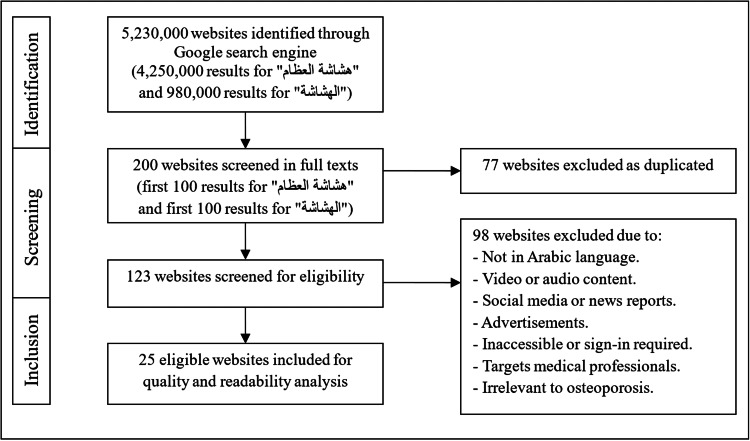
Flowchart of search results and steps of the procedure Both search terms هشاشة العظام (*hashashat aleizam*) and الهشاشة (*al hashshah*) are Arabic terms for osteoporosis.

The first 100 search results for each of the two Arabic terms were collected to be assessed by the reviewers. Duplicate websites, i.e., the websites that appeared in the search for the first term and again in the search for the second one, were assessed as a single entry. Other exclusion criteria included any non-Arabic website; any non-written content; any advertised website; any social media website; any inaccessible website; any website that required payment or registration; and any website that targeted medical professionals rather than patients. We included websites that exclusively covered osteoporosis for patient education. The included websites were classified into six categories: 1) hospitals, medical centers, and physicians; (2) health portals (websites that present health-related information on different topics); (3) commercial (websites that sell products or services for profit); (4) governmental; (5) university and academic; (6) blogs and webpages. The different stages of the search strategy that we followed are depicted in Figure 1, and the included websites are listed in Table 1.

**Table 1:** List of included websites covering osteoporosis in Arabic

Number	URL	Website name	Website type
1	https://altibbi.com	Altibbi	Hospital/medical center/physicians
2	https://www.mayoclinic.org	Mayo Clinic	Hospital/medical center/physicians
3	https://www.webteb.com	WebTeb	Health portals
4	https://www.moh.gov.sa	Saudi Ministry of Health	Governmental
5	https://www.msdmanuals.com	MSD	Hospital/medical center/physicians
6	https://www.medcare.ae	Medcare	Hospital/medical center/physicians
7	https://www.andalusiaegypt.com	Andalusia hospitals	Hospital/medical center/physicians
8	https://www.moh.gov.bh	Bahrain ministry of health	Governmental
9	https://mawdoo3.com	Mawdoo3	Blog/webpage
10	https://www.magrabi.com.sa	Magrabi hospitals	Hospital/medical center/physicians
11	https://www.topdoctors.com.sa	Top Doctors	Hospital/medical center/physicians
12	https://tebcan.com	Tebcan	Health portal
13	https://www.novomed.com	Novomed	Hospital/medical center/physicians
14	https://www.mediclinic.ae	Mediclinic	Hospital/medical center/physicians
15	https://www.tibyana.com	Tibyana	commercial
16	https://dratacan.com	Dr. Ata Can	Hospital/medical center/physicians
17	https://hip-knee.com	Hip Knee Clinic	Hospital/medical center/physicians
18	https://www.elconsolto.com	Elconsolto	Health portal
19	https://www.su.edu.sa	Shaqra university	University/academic
20	https://ahmc.com.sa	Al Haramain Medical Complex	Hospital/medical center/physicians
21	https://dr-ibrahimgado.com	Joint Clinic	Hospital/medical center/physicians
22	https://elgeaditraumatologia.com	Elgeadi Traumatologia	Hospital/medical center/physicians
23	https://globalcarehospital.com	Global Care Hospital	Hospital/medical center/physicians
24	https://www.apolloclinic.com	Apollo Clinic	Hospital/medical center/physicians
25	https://www.vejthani.com	Veithani Hospital	Hospital/medical center/physicians

The quality of the included websites was evaluated using DISCERN [16] and the Journal of the American Medical Association (JAMA) benchmarks [17], both of which are well-established tools. The Health on the Net Foundation Code of Conduct (HONcode) was not utilized in the assessment as their program was permanently discontinued, and their certificate is no longer valid.

The DISCERN tool consists of 16 questions that evaluate the reliability and quality of patient-targeted educational material. We followed the instructions of this tool, and each question was rated as an answer. The ratings are as follows: one point means the website content did not answer the question at all; two to three points mean the website partially answered the question; and five points mean the website answered the question exactly as instructed by the tool. There is no right and wrong answer to the questions of this tool, but rather a measurement of how compliant the text is with the instrument. The total score ranges from 16, the lowest possible score, to 80, the maximum possible score. Low-quality scores range from 16 to 32, moderate-quality falls between 33 and 64, and high-quality scores are 65 or above [18].

The JAMA benchmarks tool assesses the following four principles: 1) authorship (authors' names, affiliations, and credentials are mentioned); 2) attribution (sources are provided); 3) disclosure (ownership and conflicts of interest are disclosed); and 4) currency (date of posting or updating of the content is clear). Each principle is assigned one point, meaning the website’s total score may range from zero (did not comply with any of the criteria) to four (followed the four standards). The score reflects the website’s reliability, as three points or higher indicate high reliability, while lower scores imply low reliability [17].

The readability of the website content was measured using a readability calculator instrument (http://www.online-utility.org/english/readability_test_and_improve.jsp) that has been validated and employed in former studies to assess Arabic texts [18,19]. The text was evaluated using the Flesch Kincaid grade level (FKGL), Simple Measure of Gobbledygook (SMOG), and Flesch Reading Ease (FRE) scale. To be considered acceptable in terms of readability, content should score above 60 on the FRE scale and below six on both the FKGL and the SMOG scales [20].

In line with a previous study by Yurdakul et al., who evaluated English websites covering osteoporosis, the included websites that appeared on the first page of Google search results were considered to be the most viewed websites. Therefore, they were grouped under the category “first page” and compared to websites from “other pages” [12].

The quality assessment using DISCERN and JAMA was conducted by two authors (D.M.A. and M.A.A.) individually. Inter-rater agreement was determined by the interclass correlation coefficient. After that, any discrepancies in the evaluation were discussed to reach a consensus. Descriptive statistical methods were used to assess the collected data; means and standard deviations were calculated; frequencies and proportions were displayed in tables. The correlations between the quality, reliability, and readability of the websites were assessed. All data were managed and analyzed using Microsoft® Excel® 365 (Microsoft Corp., Redmond, WA) and IBM SPSS Statistics for Windows, version 27.0 (IBM Corp., Armonk, NY). P-values below 0.05 were considered to be significant.

## Results

Twenty-five websites covering osteoporosis in Arabic were evaluated in this study. Out of these, seven (28%) websites were included from the first page of Google results, and 18 (72%) websites were included from other pages. Among these websites, 17 (68%) were owned by hospitals or physicians, three (12%) were health portals, two (8%) were governmental websites, one (4%) was a commercial website, one (4%) was an academic website, and one (4%) was a blog. Table 2 shows the content characteristics of each website. The majority of websites (n = 23, 92%) discussed the causes and risk factors of osteoporosis, yet only one website (4%) covered the complications.

**Table 2:** Aspects of the disease covered by the included websites 1 = The website covered the mentioned aspect; 0 = The website did not discuss the mentioned aspect Data are presented as frequencies (n) and proportions (%).

Website	Definition	Causes and risk factors	Signs and symptoms	Diagnosis	Treatment	Complications	Prevention
1	1	1	1	1	1	0	0
2	1	1	1	1	1	0	0
3	1	1	1	1	1	0	0
4	1	1	1	1	1	0	1
5	1	1	1	1	1	0	1
6	1	1	1	1	1	0	0
7	1	1	1	1	1	0	1
8	1	1	1	0	1	0	1
9	1	1	1	1	1	0	1
10	1	1	1	0	0	0	0
11	1	1	1	1	1	0	1
12	1	1	1	0	1	0	0
13	1	1	1	1	1	0	0
14	0	1	1	1	0	0	1
15	1	0	1	1	0	0	0
16	1	0	1	0	1	0	1
17	1	1	1	1	1	0	1
18	0	1	1	1	1	0	1
19	1	1	1	1	1	0	1
20	0	1	1	0	1	0	1
21	0	1	0	1	1	1	1
22	1	1	1	1	0	0	1
23	1	1	1	1	1	0	0
24	1	1	1	1	1	0	1
25	1	1	0	1	1	0	1
Total n (%)	21 (84%)	23 (92%)	23 (92%)	20 (80%)	21 (84%)	1 (4%)	16 (64%)

**Figure 2:**
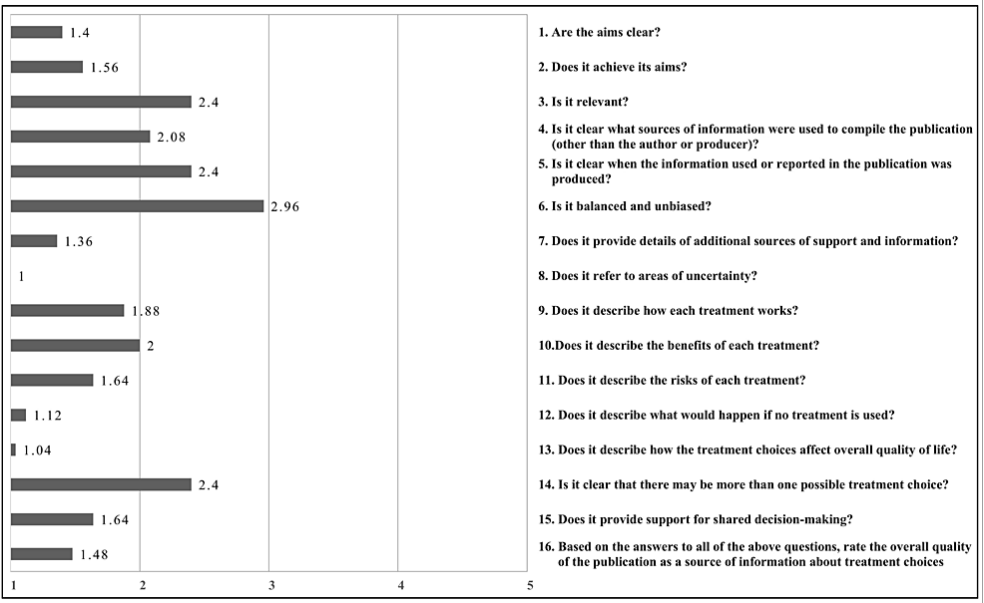
Average score for each DISCERN question Data are presented as means.

Inter-rater reliability was found to be 0.913 using the interclass correlation coefficient, with no significant differences between the two raters (p = 0.463). The overall mean DISCERN score was 28.36±12.18. The highest total score was 55 out of a maximum of 80 total scores, while the lowest total score in our study was 16, which is the lowest possible score on DISCERN. The DISCERN item with the highest average score (2.96 out of five) was about unbiased content. Meanwhile, the item with the lowest average score (one out of five) was about areas of uncertainty, as none of the websites (n = 0, 0%) discussed the gray areas and gaps of knowledge. According to DISCERN ratings, 17 (68%) websites were of low quality, eight (32%) websites were of moderate quality, and no websites were considered to be of high quality. Figure 2 shows the average ratings for each of the DISCERN questions.

**Figure 3:**
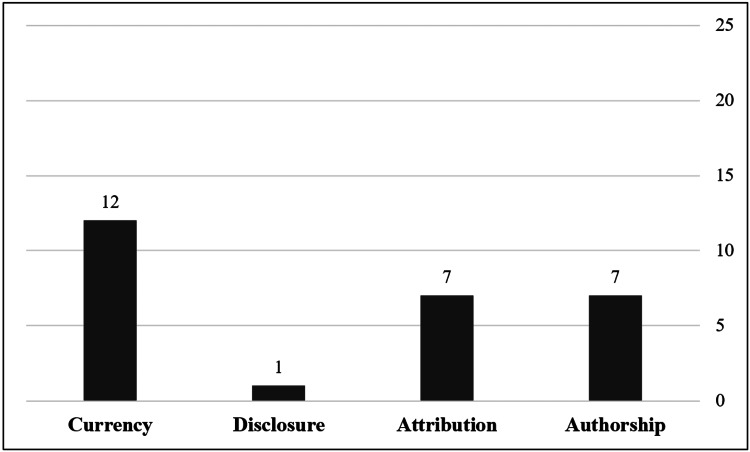
Number of websites that fulfilled each JAMA criterion Data are presented as (N). JAMA: Journal of the American Medical Association

The mean JAMA score of all websites was 1.05±1.15, with only one (4%) website scoring four total marks, while 10 (40%) websites scored 0 marks. According to JAMA scores, 22 (88%) websites had low reliability, while 3 (12%) websites had high reliability. The JAMA benchmark that was the most commonly met by websites (n = 12, 48%) was currency, while disclosure was only met by one (4%) website. Figure 3 shows the number of websites that fulfilled each criterion.

The correlation coefficient between the DISCERN and JAMA scores was determined to be 0.551.

The readability scores of all websites were, on average, 50.71±21.96 on the FRE scale, 9.25±4.89 on the FKGL, and 9.74±2.94 on the SMOG. Only seven (28%) websites were considered to have an acceptable readability score on the FRE scale, and only four (16%) websites were acceptable on the FKGL and the SMOG. The correlation coefficient between the DISCERN score and FRE score, the FKGL, and the SMOG was determined to be 0.353, -0.252, and -0.300, respectively. There were no significant differences (P = 0.215, 0.374, and 0.549) in the FRE score, the FKGL, or the SMOG, respectively, between the moderate- and low-quality websites. The correlation coefficient between the JAMA score and FRE score, the FKGL, and the SMOG was found to be -0.033, 0.146, and 0.144, respectively. There were no significant differences (P = 0.906, 0.906, and 0.906) in the FRE score, the FKGL, or the SMOG, respectively, between the high- and low-reliability websites. Table 3 shows the difference between the DISCERN score, the JAMA score, the FRE score, the FKGL, and the SMOG across the two groups of websites.

**Table 3:** Comparison between websites of the first page and other pages * Significant at P<0.05 level. n: number of websites in the category. SD: standard deviation; JAMA: Journal of the American Medical Association; FRE: Flesch Reading Ease; FKGL: Flesch-Kincaid Grade Level; SMOG: Simple Measure of Gobbledygook

Mean score (SD)	Websites on the first page n = 7	Websites on other pages n = 18	P-value
Mean JAMA score (SD)	37.71 (13.87)	24.72 (9.57)	0.004*
Mean FRE score (SD)	1.86 (1.35)	0.78 (0.94)	0.032*
Mean FKGL (SD)	50.77 (20.71)	50.70 (23.01)	0.465
Mean SMOG (SD)	9.02 (3.56)	9.35 (5.40)	0.229
Mean DISCERN score (SD)	9.76 (2.02)	9.72 (3.28)	0.308

## Discussion

Our study aimed to evaluate the quality and readability of websites covering osteoporosis in Arabic. Our assessment using the DISCERN tool yielded a mean score of 28.36±12.18 out of 80, suggesting low overall quality. The mean score was lower than in other articles that have been published. For instance, a study conducted by Wallace et al. (2005) [10] that evaluated osteoporosis educational materials online found an average DISCERN score of 35.7±18.0. None of the 25 evaluated websites were of high quality, with only eight being of moderate quality and the remaining 17 being of low quality. These low scores can be attributed to websites lacking crucial details regarding treatment instructions and options, as well as a lack of information on possible complications and side effects.

The JAMA score in our study indicates that most of these websites are unreliable, with an average score of 1.05±1.15 out of four. Only one website scored four marks on the JAMA score, while 10 websites scored 0 marks, rendering 22 (88%) websites out of the total 25 reviewed to be of low reliability. Yurdakul et al. (2021) [12] found 62.3% of websites covering osteoporosis to be of low reliability and had a total JAMA score of 2.2±1.19, which is more reliable than the current findings in our study. A more recent study by Hidayat et al. (2022) [13] analyzed the readability and quality of web-based material on osteoporotic vertebral fractures and reported a mean JAMA score of 2.21, almost the same as what Yurdakul et al. (2021) [12] had found. It is reasonable to assume that the low ratings are due to the content of JAMA benchmark criteria (authorship, attributions, disclosure, and currency), which are more often found on academic or specialized websites [11].

Assessing the readability of all the websites, they scored, on average, 50.71±21.96 on the FRE scale, 9.25±4.89 on FKGL, and 9.74±2.94 on SMOG. Seven websites were deemed acceptable on the FRE scale, while only four websites were deemed acceptable on the FKGL and SMOG tests. These results indicate that, overall, most of the websites assessed are written at a higher reading level than the acceptable reading scores [20]. Our study findings are consistent with Yurdakul et al.’s (2021), which found that many websites do not fulfill the suggested reading criteria for patient education materials, with a mean FKG score of 8.81±2.21 [12]. Moreover, Wallace et al. (2005) noted that the majority of websites have material written at a level that is higher than most adults' literacy abilities [10]. Lastly, Hidayat et al. found that the average FRE scale was 49.26 and the average FKGL was 8.38, exceeding the American Medical Association's required sixth-grade reading level [13]. As those studies all share the same findings as ours, we assume that those who write educational material for the public do not take into consideration the readability level of the readers.

Websites on the search engine's first page scored significantly higher on the DISCERN and JAMA instruments than those on other pages. This shows that search results on the first page are often of higher quality and more reliable than results on subsequent pages. There was no significant difference in scores for readability on FRE, FKGL, and SMOG. Similarly, Yurdakul et al. found that the FKG and SMOG readability scores were similar on the first 10 pages and the remaining pages, even though they did not report any difference in JAMA scores between the first 10 websites and the remaining websites [12].

Limitations and recommendations

While our study’s strength relies on using well-established tools to assess quality and readability, it is not without its limitations. First, we only used a single search engine (Google) without exploring other available search engines. Therefore, we recommend that future studies use multiple search engines with more keywords to account for different users’ access to information. Another limitation is depending on the JAMA scoring system for reliability assessment, as this scale does not consider the content of the website. Despite some websites containing high-quality information, they may present with lower scores on the JAMA evaluation. Finally, although we assessed the quality, reliability, and readability of the content present on all websites, the accuracy of the information provided was not assessed and could be subject to evaluation in future studies. Future studies should investigate techniques for improving the standards and accessibility of online health information, as well as the impact these improvements have on patient outcomes.

## Conclusions

The study highlights the importance of enhancing the quality and readability of Arabic health websites related to osteoporosis. It's crucial for healthcare professionals and website developers to work together to ensure that patients can easily access reliable and comprehensive information. This will greatly support patient education and decision-making processes. Websites targeting patients and the general public should use simple, easily understandable language that adheres to the recommended readability levels. Furthermore, since adequate knowledge is important for patients with preventable causes of osteoporosis, websites covering the disease should all discuss prevention and management.
